# Small molecule inhibitors of mammalian GSK-3β promote *in vitro* plant cell reprogramming and somatic embryogenesis in crop and forest species

**DOI:** 10.1093/jxb/erab365

**Published:** 2021-08-02

**Authors:** Eduardo Berenguer, Elena Carneros, Yolanda Pérez-Pérez, Carmen Gil, Ana Martínez, Pilar S Testillano

**Affiliations:** 1 Pollen Biotechnology of Crop Plants group, Margarita Salas Center of Biological Research, CIB-CSIC, Ramiro de Maeztu 9, 28040 Madrid, Spain; 2 Translational Medicinal and Biological Chemistry group, Margarita Salas Center of Biological Research, CIB-CSIC, Ramiro de Maeztu 9, 28040 Madrid, Spain

**Keywords:** Barley, brassinosteroids, cell reprogramming, cork oak, glycogen synthase kinase, microspore embryogenesis, small molecule inhibitors, somatic embryogenesis, rapeseed

## Abstract

Plant *in vitro* regeneration systems, such as somatic embryogenesis, are essential in breeding; they permit propagation of elite genotypes, production of doubled-haploids, and regeneration of whole plants from gene editing or transformation events. However, in many crop and forest species, somatic embryogenesis is highly inefficient. We report a new strategy to improve *in vitro* embryogenesis using synthetic small molecule inhibitors of mammalian glycogen synthase kinase 3β (GSK-3β), never used in plants. These inhibitors increased *in vitro* embryo production in three different systems and species, microspore embryogenesis of *Brassica napus* and *Hordeum vulgare*, and somatic embryogenesis of *Quercus suber*. TDZD-8, a representative compound of the molecules tested, inhibited GSK-3 activity in microspore cultures, and increased expression of embryogenesis genes *FUS3*, *LEC2*, and *AGL15*. Plant GSK-3 kinase BIN2 is a master regulator of brassinosteroid (BR) signalling. During microspore embryogenesis, BR biosynthesis and signalling genes *CPD*, *GSK-3-BIN2*, *BES1*, and *BZR1* were up-regulated and the *BAS1* catabolic gene was repressed, indicating activation of the BR pathway. TDZD-8 increased expression of BR signalling elements, mimicking BR effects. The findings support that the small molecule inhibitors promoted somatic embryogenesis by activating the BR pathway, opening up the way for new strategies using GSK-3β inhibitors that could be extended to other species.

## Teaser

Inhibition of GSK-3 activity by novel small molecules enhances somatic embryogenesis efficiency by activating the brassinosteroid pathway, an innovation with high potential to improve *in vitro* plant regeneration for crop and forest breeding techniques.

## Introduction

Agriculture in the 21st century faces significant pressure worldwide for more efficient and accelerated breeding due to population growth, climatic change, resource scarcity, and societal biosafety demands. The increasing demand for global food security in the face of a warming climate is leading researchers to investigate the physiological and molecular responses of crop plants to environmental stress, as well as to develop efficient and rapid methods to obtain new crop varieties, with increasing yield and better adapted to new environmental conditions. In this context, efficient technologies to exploit plant cell *in vitro* reprogramming potential for production of doubled-haploids (DHs), for regeneration and propagation of selected plants, and for conducting gene editing and transformation techniques (which require more efficient plant regeneration methods), are needed in plant breeding and biotechnology, as well as in applied and basic plant research.

*In vitro* embryogenesis is a fascinating example of plant cell reprogramming and totipotency, as different kinds of somatic cells, either haploids or diploids, can be reprogrammed, giving rise to an entire embryo and ultimately a plant, without the fusion of gametes (Fehér, 2015; Germanà and Lambardi, 2016; Loyola-Vargas and Ochoa-Alejo, 2016). In the case of stress-induced microspore embryogenesis, the microspore (haploid cell, precursor of the pollen grain) is reprogrammed towards an embryogenic pathway by stress treatment (Touraev *et al.*, 1996; Maluszynski *et al.*, 2003; Dwivedi *et al.*, 2015; Testillano, 2019). The resulting haploid embryo, after spontaneous or chemically induced diploidization, will produce DH plants, which are widely used by seed and nursing companies to accelerate breeding programmes. While classical genetic breeding strategies to improve traits in crops require many generations and numerous selection processes to produce new varieties, DH plants are unique sources of new genetic variability, fully homozygous for each locus, and fixed in only one generation (Maluszynski *et al.*, 2003; Touraev *et al.*, 2009; Germanà, 2011; Testillano, 2019). When cells from other tissues are reprogrammed to embryogenesis, somatic embryogenesis has proved to be very useful for propagation of species with long reproductive cycles or low seed set in a large variety of crop and forest species (Loyola-Vargas and Ochoa-Alejo, 2016; Díaz-Sala, 2018; Pais, 2019), due to its great potential for large-scale clonal propagation and cryopreservation of elite genotypes, as well as for production of genetically modified and, more recently, gene-edited plants with improved traits.

However, in many crop and forest species, it is challenging to find efficient conditions for *in vitro* cell reprogramming and regeneration from somatic cells, either microspores, protoplasts, or cells from vegetative or immature embryo tissues. Although somatic embryogenesis is currently widely exploited, it is still highly, or even completely, inefficient in many plants of economic interest. The induction of somatic embryogenesis is a multifactorial developmental process that is initiated in response to exogenous stimuli, usually a stress treatment. Knowledge gained in recent years has revealed that initiation and progression of somatic embryogenesis involve a complex network of factors, whose roles are not yet well understood (Horstman *et al.*, 2017; Testillano, 2019; Ibañez *et al.*, 2020).

Advances in chemically controlled reprogramming of specialized mammalian cells into pluripotent cells have demonstrated the enormous potential of application of cell-permeable synthetic small molecules to regulate cellular reprogramming (Ma *et al.*, 2017; Kim *et al.*, 2020). To date, numerous chemical libraries of small molecules have been developed, and their screening has identified different synthetic compounds that efficiently induce cell reprogramming (Tang and Cheng, 2017). Although the molecular mechanisms of most cell reprogramming processes and how small molecules mediate cell fate transition are largely unknown, there are many small molecules that have proven reprogramming effects *in vitro* in mammalian cells, some of which are epigenetic modulators and inhibitors of key enzymatic activities (Kim *et al.*, 2020). Also in plant biology research, chemical approaches have shown enormous potential to decipher molecular pathways (Hicks and Raikhel, 2012; Chuprov-Netochin *et al.*, 2016); a number of studies have reported the identification of physiologically active compounds via phenotypic screening of chemical libraries, studies that have targeted a few plant-specific processes of interest (Hicks and Raikhel, 2012; Dejonghe and Russinova, 2014; Dauphinee *et al.*, 2019). Furthermore, the use of small molecules, particularly some epigenetic inhibitors, has recently been demonstrated to promote *in vitro* plant cell reprogramming in microspore embryogenesis (Li *et al.*, 2014; Solís *et al.*, 2015; Berenguer *et al.*, 2017); however, their efficient application in plant biotechnology approaches is still a challenge.

Interestingly, one of the groups of small molecules with reported effects in reprogramming of various mammal somatic cells is the inhibitor of glycogen synthase kinase 3β (GSK-3β; Ma *et al.*, 2017). Several studies have reported the discovery of new small compounds that inhibit mammalian GSK-3β activity and their use in the reprogramming of human stem cells and *in vivo* enhancement of neurogenesis (Martínez *et al.*, 2002; Morales-García *et al*., 2012). In mammalians, GSK-3 exists as two isoforms, α and β, whereas in plants there is a large multigene family; however, all plant GSK-3-like kinases have a very similar structure to GSK-3β in the catalytic domain (Saidi *et al.*, 2012). Plant GSK-3-like or Shaggy-like kinases are actively implicated in hormonal signalling networks during development and stress responses (Youn and Kim, 2015). In Arabidopsis, signal transduction of the hormone brassinosteroid (BR) involves a gene named *BRASSINOSTEROID-INSENSITIVE 2* (*BIN2*), which encodes a GSK-3 kinase. The Arabidopsis genome encodes 10 GSK-3-like kinases that are clustered into groups I–IV, where BIN2 is one of the three members in group II. Despite functional redundancy, BIN2 plays a dominant role among the three group II members in regulating BR signalling (Yan *et al.*, 2009). BRs are polyhydroxylated plant steroid hormones perceived on the plasma membrane by the leucine-rich repeat receptor kinase BRI1 (BR-INSENSITIVE 1), its two homologues BRL1 (BRI1-LIKE1) and BRL3 (BRI1-LIKE3), and the co-receptor SERK3/BAK1 (SOMATIC EMBRYOGENESIS RECEPTOR KINASE 3/BRI1-ASSOCIATED KINASE 1) (Li and Chory, 1997; Caño-Delgado *et al.*, 2004; Albrecht *et al.*, 2008). The kinase signalling cascade through BRI1/BAK1 activates BSU (BRI1 SUPRESSOR) to inhibit the GSK-3 kinase BIN2, a negative regulator of the BR pathway (Yun *et al.*, 2009). In the absence of BR, BIN2 phosphorylates numerous substrates including BES1 (BRI1-EMS-SUPPRESSOR 1) and BZR1 (BRASSINAZOLE-RESISTANT 1), promoting their cytoplasmic retention, inhibiting their DNA binding activity, and stimulating their degradation (Nolan *et al.*, 2020). BES1/BZR1 family transcription factors are master components of the BR pathway through regulation of the expression of numerous genes for BR response, as well as BR biosynthetic genes *CPD* (*CONSTITUTIVE PHOTOMORPHOGENESIS AND DWARFISM*) and *DWF4* (*DWARF 4*), and BR catabolic genes such as *BAS1* (*PHYB ACTIVATION-TAGGED SUPPRESSOR 1*) (He *et al.*, 2005; Nolan *et al.*, 2020). Thus, the BR signalling pathway depends on BR levels, the protein phosphorylation cascade driven by BIN2, protein degradation, and downstream transcriptional regulation.

The induction of most somatic embryogenesis systems, except for microspore embryogenesis, is usually triggered by exogenous hormones, mostly by the synthetic auxin 2,4-dichlorophenoxyacetic acid (2,4-D), in combination or not with stress treatments; after induction, auxin-free medium is required to initiate embryogenesis (Fehér, 2015; Díaz-Sala, 2018). Microspore embryogenesis systems do not require exogenous auxin; a transient stress treatment is enough to induce the change of cell fate towards embryogenesis. After induction, endogenous auxin biosynthesis, signalling, and polar transport are activated and required for somatic embryogenesis initiation and progression (Rodríguez-Sanz *et al.*, 2015; Pérez-Pérez *et al.*, 2019). With the exception of auxin, very scarce information is available on the role in somatic embryogenesis of other endogenous hormones, such as BRs.

In this work, we have evaluated the effect on *in vitro* embryogenesis of four small heterocyclic molecules, inhibitors of mammal GSK-3β, with different chemical structures and binding modes: TDZD-8 binds to GSK-3β in a covalent reversible manner (Martínez *et al.*, 2002); VP3.15 is a substrate competitive inhibitor (Palomo *et al.*, 2012); VP0.7 modulates the kinase in an allosteric fashion (Palomo *et al.*, 2011); and finally VP3.36 is an ATP competitive inhibitor (Pérez *et al*., 2011). The compounds have been tested in three different somatic embryogenesis systems of crop and forest species, namely in microspore embryogenesis of *Brassica napus* (rapeseed) and *Hordeum vulgare* (barley), and somatic embryogenesis of *Quercus suber* (cork oak). The results showed that treatments with these compounds were able to enhance embryogenesis initiation efficiency and embryo production in all the *in vitro* systems tested. Further analyses in *B. napus* with the small molecule TDZD-8 indicated that it increased expression of embryogenesis-specific genes, inhibited GSK-3 activity, and activated BR signalling in microspore cultures.

## Materials and methods

### Plant material and growth conditions

Plants of *Brassica napus* L. (rapeseed) cv. ‘Topas’ line DH407 and *Hordeum vulgare* L. (barley) cv. ‘Igri’ were used as donor plants for isolated microspore cultures. Rapeseed seeds were germinated and grew in growth chambers under controlled conditions, as previously described (Berenguer *et al.*, 2020). Barley seeds were vernalized, germinated, and grew in a greenhouse, under controlled conditions as reported (Pérez-Pérez *et al.*, 2019).

Plant material of *Quercus suber* L. (cork oak) trees was collected in the countryside (El Pardo region, Madrid, Spain) during August–September. Branches with several catkins, containing immature zygotic embryos, were excised, kept at 4 °C for several days, and used for somatic embryogenesis cultures, as described (Testillano *et al.*, 2018).

### *In vitro* embryogenesis cultures from isolated microspores and immature zygotic embryos

For *B. napus* and *H. vulgare* microspore embryogenesis induction, isolated microspore cultures were kept in liquid media (NLN-13 for *B. napus*, KBP for *H. vulgare*), and embryogenesis was induced by stress treatments of 32 °C and 4 °C, respectively, as reported for both species (Prem *et al.*, 2012; Rodríguez-Serrano *et al.*, 2012). For rapeseed microspore culture, flower buds with microspores at the vacuolated stage (the most responsive stage for embryogenesis induction) were sterilized and crushed in a cold mortar with 5 ml of cold NLN-13 medium containing 13% sucrose. The suspension was filtered through a 48 μm nylon filter and made up to a volume of 10 ml with NLN-13 medium. The filtrate was then centrifuged at 1100 rpm for 5 min at 4 °C, and the pellet was resuspended in 10 ml of cold NLN-13 and centrifuged again, repeating three times for washing. The final pellet was suspended in NLN-13, and the cell density was adjusted to 10 000 cells ml^–1^. Isolated microspore cultures were subjected to a temperature of 32 °C for induction of embryogenesis. Around 10 d after culture initiation, when globular embryos were observed, cultures were shifted to 25 °C on a gyratory shaker at 60 rpm until complete development of embryos was observed. For barley microspores, spikes containing microspores at the vacuolated stage were collected, surface-sterilized, and treated at 4 °C for 23–24 d. To isolate the microspores, the spikes were blended in 20 ml of pre-cooled 0.4 M mannitol using a Waring blender (Eberbach, Ann Arbor, MI, USA), and the extract was filtered through a 100 µm nylon mesh. The microspore suspension collected was transferred into a 50 ml tube and centrifuged at 100 *g* for 10 min at 4 °C, and the pellet was resuspended in 8 ml of ice-cold 0.55 M maltose. This volume was distributed between two 15 ml tubes and each aliquot was carefullly overlayered with 1.5 ml of mannitol solution. After gradient centrifugation at 100 *g* for 10 min at 4 °C, the interphase band consisting of an enriched population of vacuolated microspores was resuspended in mannitol solution, giving a final volume of 20 ml. The pelleted microspores were diluted in an appropriate volume of KBP medium to obtain a cell density of 1.1×10^5^ cells ml^–1^. The microspores were incubated at 25 °C in the dark. After ~30 d in culture, cotyledonary (in *B. napus*) and coleoptilar (in *H. vulgare*) embryos were formed, similar to zygotic embryogenesis in dicot and monocotyledoneous species.

For somatic embryogenesis of *Q. suber*, immature acorns at the responsive stage of early cotyledonary embryos (immature zygotic embryos) were cultivated in induction medium [Murashige and Skoog (MS) micronutrients and vitamins, Sommer macronutrients, 0.5 g l^–1^ glutamine, 30 g l^–1^ sucrose, 8 g l^–1^ agar], containing 0.5 mg l^–1^ 2,4-D at 25 °C with 16/8 h light/darkness for 1 month and then transferred to a regulator-free medium (renewed every 30 d), where embryogenic masses arise and proliferate, and somatic embryos were formed by indirect and recurrent embryogenesis (Testillano *et al.*, 2018).

### Small molecule inhibitors of GSK-3β

The four GSK-3β inhibitors used for the treatments were TDZD-8 (mol. wt 222 Da), VP3.15 (mol. wt 528 Da), VP0.7 (mol. wt 429 Da), and VP3.36 (mol. wt 268 Da). Their chemical structure and inhibitory properties have been previously reported; their IC_50_ values of human recombinant GSK-3β are: 2 µM for TDZD-8, 1.6 µM for VP3.15, 3.1 µM for VP0.7, and 4.4 µM for VP3.36. They all have been synthesized in our laboratory following previously described procedures (Martínez *et al.*, 2002; Palomo *et al.*, 2011, 2012; Pérez *et al*., 2011).

### Treatments with small molecule GSK-3β inhibitors and brassinazole

Stock solutions of GSK-3β inhibitors at 10 mM, and brassinazole (BRZ) at 5 mg ml^–1^ in DMSO (Sigma-Aldrich) were used. First, assays with the four GSK-3β inhibitors TDZD-8, VP3.15, VP0.7, and VP3.36 were performed using 3-4 concentrations, ranging from 0.5 µM to 5 µM, in rapeseed microspores cultures. After evaluation of the effects on embryogenesis initiation efficiency, three selected compounds were tested in *in vitro* embryogenesis systems of barley, at similar and slightly higher concentrations, and cork oak, at 10× higher concentrations (since gelled media present lower diffusion and limited availability of their components compared with liquid media).

In microspore cultures of *B. napus* and *H. vulgare*, appropriate volumes of stock solution of each compound were added to culture plates and mixed with liquid culture medium, at the initiation of microspore culture. The BR biosynthesis inhibitor BRZ (Sigma-Aldrich) was also applied from culture initiation, at concentrations of 10 µM and 20 µM, which are concentration ranges used in previous reports for plant *in vitro* systems (Asami *et al.*, 2000).

In somatic embryogenesis cultures of *Q. suber*, small molecule inhibitors of GSK-3β were applied at 25 µM and 100 µM to isolated embryogenic masses. Appropriate volumes of stock solutions (10 mM in DMSO) of each compound were added to cooled medium, before its gelling, inside culture plates, at the initiation of culture. Embryogenic masses were cultured in medium containing the inhibitors; after 15–30 d of treatment they were transferred to a culture medium without the compound, for 30 d.

Three independent experiments were performed for each *in vitro* embryogenesis system, inhibitor, and concentration. Mock parallel plates of the same cultures, to which we added the highest volume of DMSO used among the different concentrations of inhibitors, were kept as controls.

### Evaluation of *in vitro* embryogenesis induction efficiency and embryo production

Quantification of the embryogenesis induction efficiency in *B. napus* and *H. vulgare* microspore cultures was performed as previously reported (Berenguer *et al.*, 2017). The number of proembryos, the first sign of microspore embryogenesis initiation, was quantified in control and treated cultures through randomly obtained micrographs from a stereomicroscope (Leica MZ16F) and an inverted microscope (Leica DMI6000B). Three independent experiments were performed and a minimum of 1000 proembryos were counted per each *in vitro* system and treatment.

The cellular organization of proembryos in control and treated cultures was assessed by DAPI staining to visualize nuclei, using 10 µg ml^–1^ staining solution, as described (Solís *et al.*, 2008). Squash preparations of 6-day-old proembryos were analysed under fluorescence microscopy (Carl Zeiss AG) using UV excitation for observing nuclei.

To evaluate total embryo production in control and treated cultures of microspore embryogenesis of *B. napus* and *H. vulgare*, developed and mature cotyledonary and coleoptilar embryos were quantified after 30 d and 40 d of culture, respectively. Three independent experiments were performed, and the number of total embryos per plate was counted through images captured by a Nikon D810 camera with a 60 mm f2.8 Micro-Nikkor (Nikon) lens. Both proembryo and embryo quantifications were performed using image analysis tools of Adobe Photoshop CS5.1 software. Results were normalized to mean values in control cultures.

Embryo production in somatic embryogenesis of *Q. suber* was quantified in control and treated cultures by the number of cotyledonary embryos produced per gram of embryogenic masses at culture initiation. Three independent experiments were performed.

### Germination assays of microspore embryos

To evaluate the quality of the produced embryos in control and treated cultures during microspore embryogenesis of *B. napus*, cotyledonary embryos after 30 d of culture were subjected to *in vitro* germination conditions, as described (Prem *et al.*, 2012). Embryos were air-dried on filter paper for 5–10 min, and then incubated on germination medium [MS, 2% sucrose, 7 g l^–1^ agar (w/v)] at 18 °C in darkness during 20–24 d. For plantlet conversion, germinated embryos were shifted to 25 °C, 16 h photoperiod conditions for 10 d, transferred to tubes for further growth, and finally acclimated to *ex vit*ro conditions in pots, as reported (Prem *et al.*, 2012).

### GSK-3 activity assay (Kinase-Glo luminescent assay)

GSK-3 activity assay was performed in consecutive developmental stages of *B. napus* microspore embryogenesis cultures: vacuolated microspores; proembryos; globular embryos; and cotyledonary embryos. Total proteins were extracted from *in vitro* samples ground in extraction buffer [50 mM HEPES pH 7.1, 2 mM DTT, 500 µM phenylmethylsulfonyl fluoride (PMSF), 1 mM EDTA, and 1 mM EGTA], and protein concentrations were determined by the method of Bradford (1976), and adjusted to equal concentrations for all samples, using the Bio-Rad Protein Assay (Quick-Start Bradford Dye Reagent, Bio-Rad). GSK-3 enzymatic activity assay was performed as described (Baki *et al.*, 2007; Gandini *et al.*, 2018) using the pre-phosphorylated polypeptide substrate YRRAAVPPSPSLSRHSSPHQ(psS)EDEEE (GS-2 peptide, Millipore), ATP (Sigma Aldrich), and the Kinase-Glo luminescent kinase assay (Promega).

Kinase-Glo assays were performed in assay buffer [50 mM HEPES (pH 7.1), 1 mM EDTA, 1 mM EGTA, and 15 mM magnesium acetate] using black 96-well plates. In the assay, 20 μl of assay buffer containing 25 μM substrate (GS-2 peptide) and 1 μM ATP were added to each well followed by 20 μl (10 ng) of protein extract. The enzymatic reaction was incubated for 30 min at 30 °C, then the reaction was stopped by addition of an equal volume of Kinase-Glo reagent (40 μl). After 10 min incubation at room temperature, luminescence was recorded using a Multiskan™ Sky Microplate UV/Vis Spectrophotometer (Thermo Fisher Scientific). The activity is proportional to the difference of the total and consumed ATP. The amount of ATP was proportional to the luminescent signal measured as RLUs (relative light units) and inversely correlated to GSK-3 enzymatic activity.

To evaluate the effect of TDZD-8 on GSK-3 activity in culture samples, protein extracts from microspore-derived proembryos produced after 4 d in control cultures and cultures treated with TDZD-8 (from culture initiation until the proembryo stage, i.e. 4 d) were used in the activity assay, following the same procedure as described above.

### Quantitative real-time PCR (RT-qPCR) analysis

Total RNA was extracted from *in vitro* samples using the RNeasy^®^ Plant Mini Kit (Qiagen) according to the manufacturer’s instructions and subsequently treated with RNase-free DNase using the TURBO DNA-free kit (ThermoFisher) to remove contaminating DNA. cDNAs were obtained from 1.5 μg of RNA using Superscript™ II reverse transcriptase (Invitrogen Life Technologies), and RT-qPCR analyses were performed using the FastStart DNA Green Master (Roche Diagnostics) on the iQ5 Real-Time PCR Detection System (Bio-Rad) with qPCR conditions and normalized expression as previously reported (Pérez-Pérez *et al.*, 2019).

Conditions for qPCR were as follows: initial denaturation at 95 °C for 30 s, followed by 40 cycles of 5 s at 95 °C and 30 s at 58 °C. After each run, by heating the samples from 65 °C to 95 °C, a dissociation curve was acquired to check for amplification specificity. Serial dilutions of cDNA were used to determine the efficiency curve of each primer pair. *HELICASE* (*HEL*) was used as the internal reference gene; independent amplification experiments with *HEL* showed its stable expression at the four developmental stages analysed, as well as in culture samples under control conditions and TDZD-8 treatment, which validated *HEL* as the reference gene for the analyses (Supplementary Table S1). A minimum of three biological and three technical replicates were analysed. Data were analysed with the Bio-Rad CFX Manager 3.1 (3.1.1517.0823), using the Livak calculation method (Livak and Schmittgen, 2001). Transcript levels were normalized to vacuolated microspore stage levels, when analysing different developmental stages, and to control culture samples when analysing TDZD-8-treated cultures. Differences among several developmental stages were tested by one-way ANOVA followed by Tukey test; differences between two conditions (control and treated cultures) were tested by Student’s-*t* test, in all cases at *P*≤0.05.

The sequences of the genes *BRASSINOSTEROID-INSENSITIVE2* (*BnBIN2*), *BRASSINAZOLE-RESISTANT1* (*BnBZR1*), *BRI1-EMS-SUPPRESSOR1* (*BnBES1*), the BR biosynthesis gene *CONSTITUTIVE PHOTOMORPHOGENESIS AND DWARFISM* (*BnCPD*), the BR inactivation gene *PHYB ACTIVATION-TAGGED SUPPRESSOR1* (*BnBAS1*), and the auxin biosynthesis gene *TRYPTOPHAN AMINOTRANSFERASE OF ARABIDOPSIS 1* (*BnTAA1*) were selected from the *Brassica rapa* database (http://www.brassicadb.cn/). The sequence of embryogenesis-specific genes *FUSCA 3* (*BnFUS3*), *LEAFY COTYLEDON2* (*BnLEC2*), and *AGAMOUSLIKE 15* (*BnAGL15*) were selected from the GenBank database (https://www.ncbi.nlm.nih.gov/genbank). The oligonucleotides designed with Primer 3 software are listed in Supplementary Table S2.

## Results

### Effect of GSK-3β inhibitors during microspore embryogenesis of *Brassica napus*

**Fig. 1.**
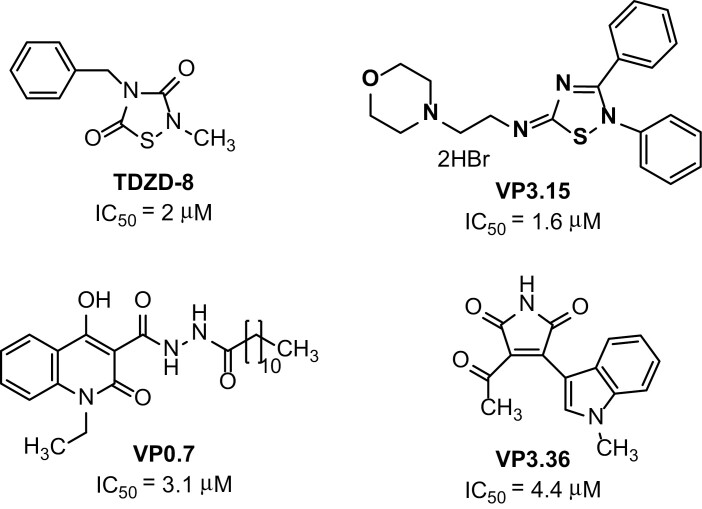
Molecular structure of the small molecule inhibitors of mammalian GSK-3β. The compounds are named TDZD-8, VP3.15, VP0.7, and VP3.36. The inhibitory potency of each compound is indicated by their IC_50_ values which are the concentration required to inhibit, *in vitro*, 50% of the human GSK-3 enzymatic activity.

Four small heterocyclic compounds, TDZD-8, VP3.15, VP3.36, and VP0.7, with different chemical structures (Fig. 1), that had been proved to inhibit mammalian GSK-3β activity (Martínez *et al.*, 2002; Palomo *et al.*, 2011, 2012; Pérez *et al*., 2011) were initially tested in *B. napus* microspore embryogenesis, as a model system for the process of *in vitro* cell reprogramming and embryo production.

**Fig. 2.**
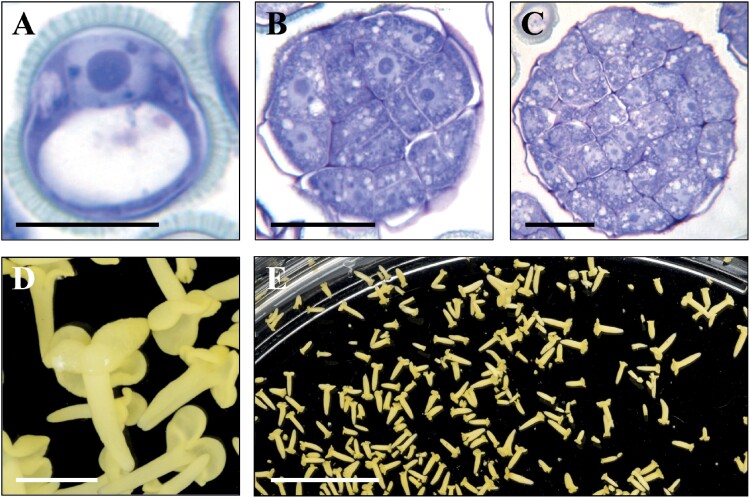
Developmental stages of microspore embryogenesis of *B. napus*. Representative micrographs of toluidine blue-stained sections of (A) isolated vacuolated microspore (culture initiation), (B) proembryo (embryogenesis initiation), (C) globular embryo, and (D, E) cotyledonary embryos. (D) Detail at higher magnification. (E) Panoramic view of a region of the culture plate. Scale bars, 10 µm in (A–C); 1 mm in (D); and 10 mm in (E).

Isolated vacuolated microspores (Fig. 2A) were cultured in liquid medium and subjected to stress treatment of 32 °C to induce *in vitro* microspore embryogenesis in *B. napus* (Prem *et al.*, 2012). After 4 d under these *in vitro* conditions, responsive microspores were reprogrammed and produced multicellular structures or proembryos (Fig. 2B). During the following days of culture, microspore embryos exhibiting the typical developmental embryogenesis stages of a dicot plant—globular (Fig. 2C), heart-shaped, and torpedo embryos—were observed. Finally, fully differentiated cotyledonary embryos were formed after 30 d in culture (Fig. 2D, E).

**Fig. 3.**
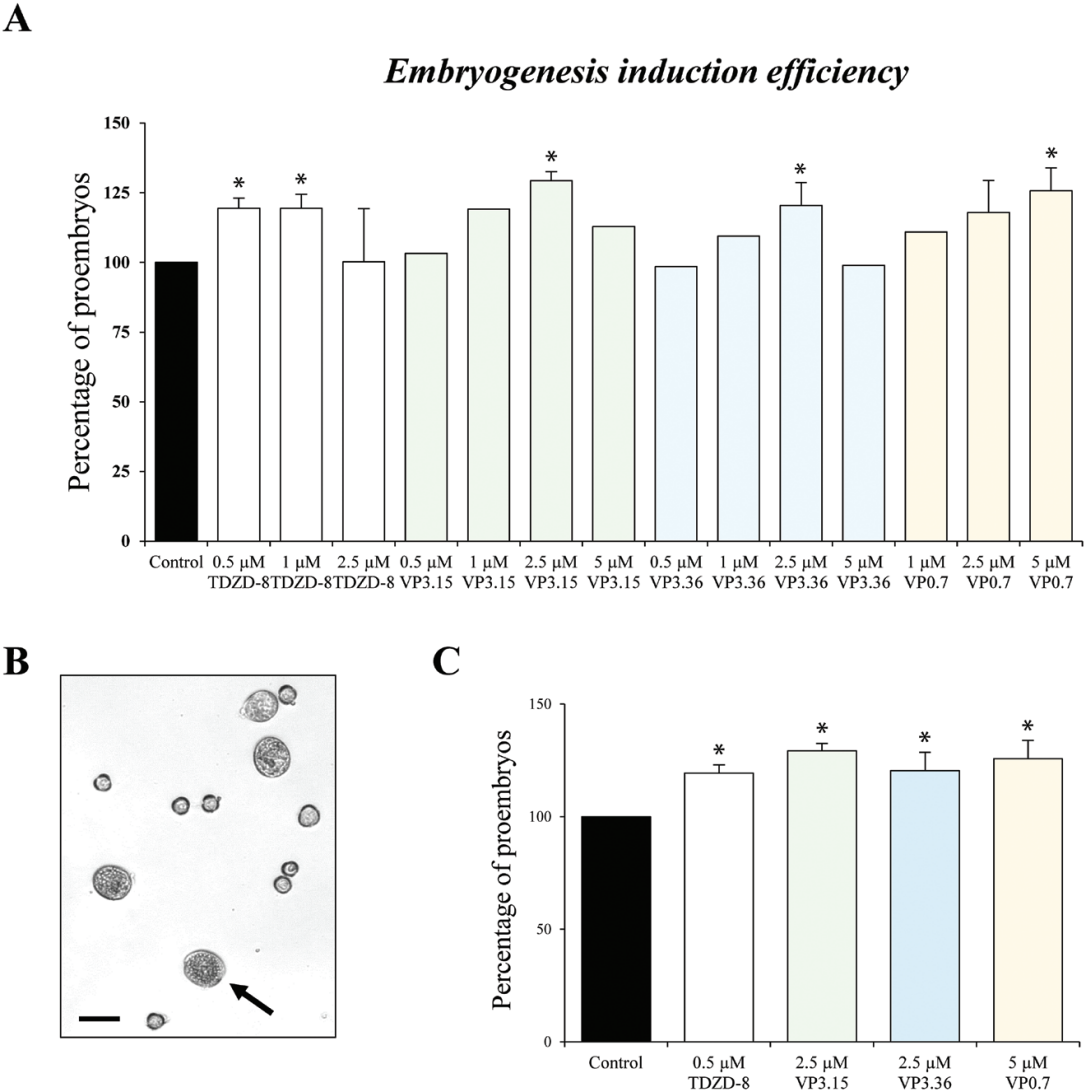
Effect of small molecule GSK-3β inhibitors on embryogenesis induction efficiency in microspore cultures of *B. napus*. (A) Histogram with the quantification of proembryos, the first sign of embryogenesis initiation, in control cultures and cultures treated with the four small molecules at different concentrations. (B) Representative micrograph of a 4 d microspore culture showing proembryos formed (indicated by arrows). Scale bar=20 µm. (C) Histogram with proembryo quantification in control and treated cultures with the selected (best) concentration of each compound. Columns represent the mean ±SEM. Values have been normalized to the control culture (100%). Asterisks in (A) and (C) indicate statistically significant differences (*P*<0.05) between control and treated cultures obtained after Student’s *t*-test.

Treatments were performed on microspore cultures with the four inhibitors at 3–4 different concentrations, from 0.5 µM to 5 µM. The efficiency of embryogenesis induction was evaluated through the frequency of proembryo formation in control and treated cultures, after 4 d of culture (Fig. 3A). Proembryos, the first morphological sign of embryogenesis initiation, were identified as multicellular structures with rounded morphology, higher density, and larger size than microspores, fully or partially surrounded by the exine (microspore wall); they could be clearly distinguished and quantified with an inverted microscope (Fig. 3B). After 4 d, we found that cultures treated with all GSK-3β inhibitors, at least at one or two of the concentrations used, led to an increase of embryogenesis induction efficiency, the proportion of proembryos being up to 20–30% higher compared with control cultures, at the best concentration for each compound (Fig. 3C), which was selected for further analyses.

**Fig. 4.**
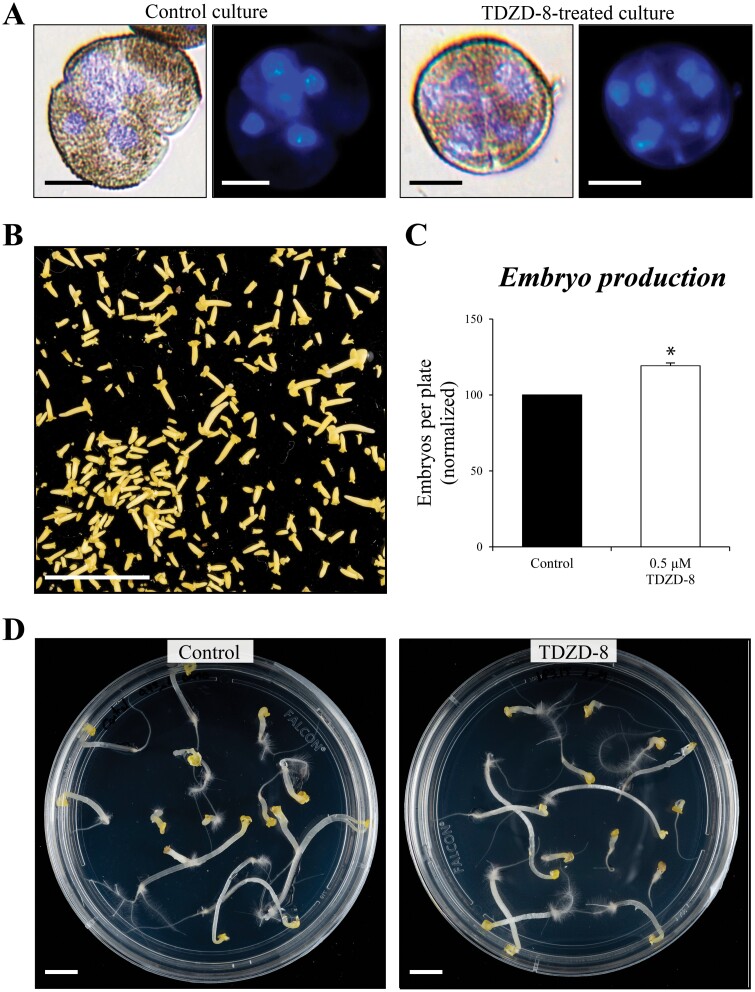
Effects of TDZD-8 on microspore embryogenesis progression in *B. napus*. (A) DAPI staining of proembryos in control and TDZD-8-treated cultures, revealing several nuclei (blue signal) in the proembryos, which indicates embryogenesis initiation. (B) Microspore culture after 30 d treatment with TDZD-8 showing predominantly cotyledonary embryos. (C) Quantification of total embryo production in control and TDZD-8-treated cultures after 30 d culture. Columns represent the mean ±SEM. Values have been normalized to the control culture (100%). Asterisks indicate statistically significant differences (*P*<0.05) between control and treated cultures by Student’s *t*-test. (D) Germinated embryos of control and TDZD-8-treated cultures. Scale bars, 10 µm in (A); and 10 mm in (B, D).

To confirm whether proembryos quantified were indeed multicellular structures containing several nuclei, we performed DAPI staining. Fluorescence microscopy analysis showed that proembryos from treated cultures contained several nuclei, like proembryos of their corresponding control culture (Fig. 4A), indicating that after reprogramming, cell division occurred similarly in control and treated cultures, and that the treatments did not affect the structural organization of proembryos.

For the subsequent analyses, we selected TDZD-8 as a representative compound with a well-documented activity as a mammalian GSK-3β inhibitor (Martínez *et al.*, 2002). To further analyse the effect of GSK-3β inhibitors on embryo development, embryo production was quantified. After 30 d, both control and treated microspore cultures showed embryos at various developmental stages, predominantly cotyledonary embryos (Fig. 4B). We quantified the total number of cotyledonary embryos produced in control and TDZD-8-treated cultures, at the concentration that produced the best effects in proembryos. As shown in Fig. 4C, embryo production was higher with TDZD-8 treatment in comparison with untreated cultures, the increase in embryos being ~20%.

To evaluate the quality of embryos produced in microspore embryogenesis cultures in the presence of the inhibitor, embryo germination assays were performed. Fully developed cotyledonary embryos from 30 d control and TDZD-8-treated cultures were desiccated and cultured under germination conditions. Embryos from treated cultures germinated very well, producing roots and hypocotyl, and in the same proportion as embryos from control cultures (Fig. 4D). Irrespective of the use of inhibitors during *in vitro* embryogenesis, all germinated embryos were able to produce *in vitro* plantlets which further acclimatized and developed into mature plants up to the flowering stage, growing in a similar way to donor plants (data not shown), as reported for microspore-derived embryos of *B. napus* (Prem *et al.*, 2012).

### Effect of the GSK-3β inhibitor TDZD-8 on overexpression of embryogenesis-specific genes during microspore embryogenesis

To gain more insight into the effect of the small molecule inhibitor TDZD-8 on microspore embryogenesis, we analysed the expression patterns of embryogenesis-specific genes. We selected the key transcription factors FUSCA 3 (FUS3), LEAFY COTYLEDON 2 (LEC2), and AGAMOUSLIKE 15 (AGL15), that have been found to be up-regulated during the induction of somatic embryogenesis in different species, including *B. napus* (Malik *et al.*, 2007; Horstman *et al.*, 2017; Méndez-Hernández *et al.*, 2019; Ibañez *et al.*, 2020). Furthermore, expression of the key enzyme of auxin biosynthesis TRYPTOPHAN AMINOTRANSFERASE OF ARABIDOPSIS 1 (TAA1) was analysed, since TAA1 up-regulation and *de novo* auxin biosynthesis are required for initiation of microspore embryogenesis (Rodríguez-Sanz *et al.*, 2015; Pérez-Pérez *et al.*, 2019).

**Fig. 5.**
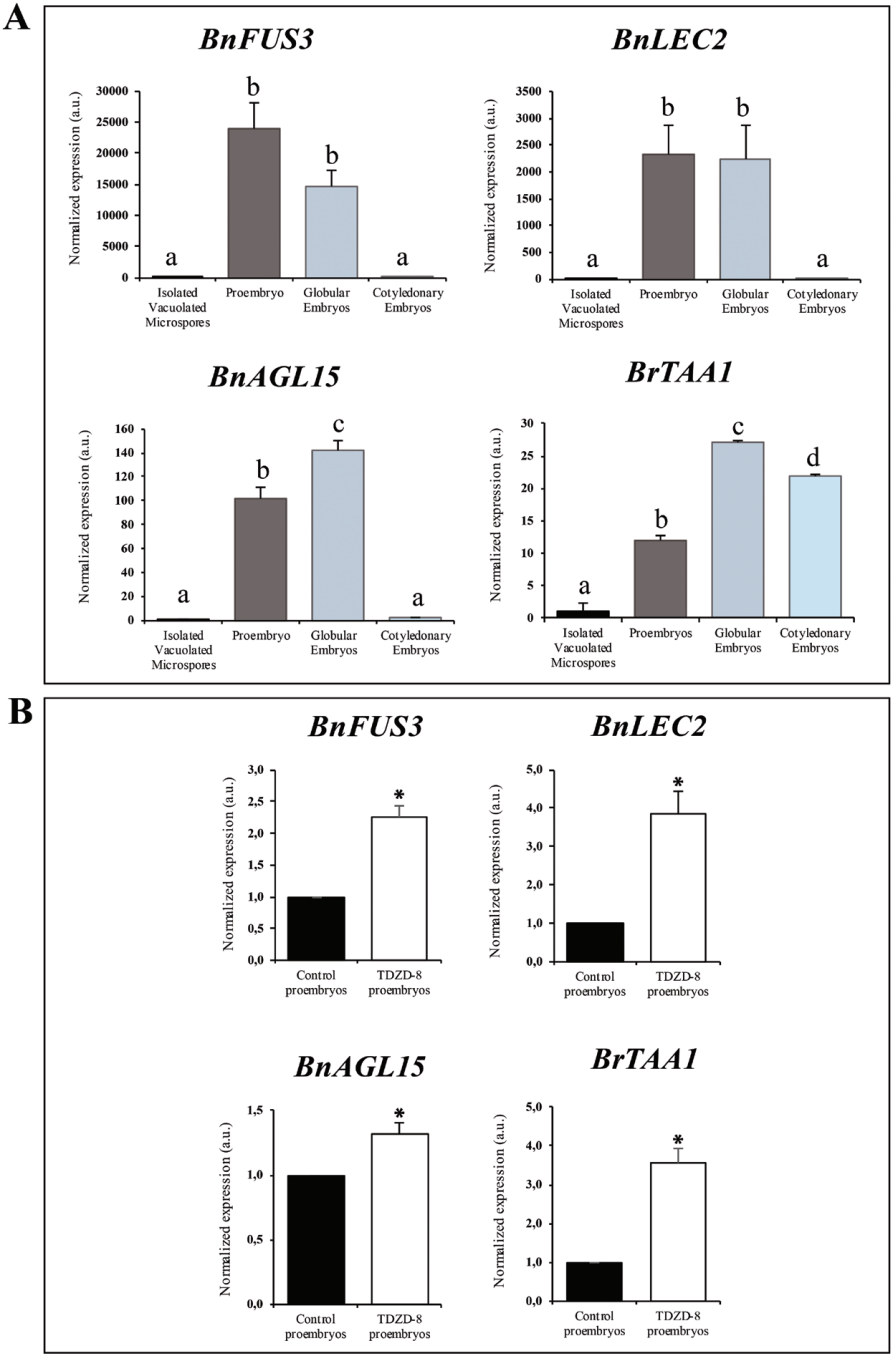
Effect of TDZD-8 on expression of embryogenesis marker genes in microspore embryogenesis of *B. napus*. (A) Expression profiles during microspore embryogenesis of *BnFUS3*, *BnLEC2*, *BnAGL15* (embryogenesis-specific genes), and *BnTAA1* (auxin biosynthesis gene) in control cultures without the inhibitor. Values are normalized to vacuolated microspore expression levels. Data represent the mean ±SEM. Different letters indicate statistically significant differences (*P*<0.05) obtained after ANOVA and subsequent Tukey HSD tests. (B) Expression of *BnFUS3*, *BnLEC2*, *BnAGL15*, and *BnTAA1* in control and TDZD-8-treated cultures at the proembryo stage. Values are normalized to control culture levels. Data represent the mean ±SEM. Asterisks indicate statistically significant differences (*P*<0.05) obtained by Student’s *t*-test.

Analyses by RT-qPCR revealed similar expression patterns for the three embryogenesis marker genes during microspore embryogenesis, in the absence of the inhibitor (Fig. 5A). *BnFUS3*, *BnLEC2*, and *BnAGL15* were not expressed in microspores at the time of culture initiation; however, gene expression was highly induced at early stages, in proembryos, and in the globular embryo stage, decreasing at advanced stages in cotyledonary embryos (Fig. 5A). The *BnTAA1* expression level was low in vacuolated microspores and increased after initiation of embryogenesis, in proembryos, and globular and cotyledonary embryos (Fig. 5A).

We compared the levels of expression in control cultures and cultures treated with TDZD-8, in proembryos, at the stage of embryogenesis initiation. In all cases, embryogenesis marker genes *BnFUS3*, *BnLEC2*, and *BnAGL15*, and the auxin biosynthesis gene *BnTAA1* showed a significant increase of expression in TDZD-8-treated cultures in comparison with control cultures (Fig. 5B), with the transcript increase being higher in *BnFUS3*, *BnLEC2*, and *BnTAA1* than in *BnAGL15.*

### GSK-3 activity during microspore embryogenesis and effect of the small molecule inhibitor TDZD-8 on this activity

Since application of small molecules that inhibited GSK-3β protein kinases in mammals enhanced embryogenesis induction efficiency in *B. napus* microspore cultures, we analysed the presence of this enzymatic activity in microspore cultures, at selected developmental stages: ‘isolated vacuolated microspores’ (before stress treatment, at the beginning of the culture), ‘proembryos’ (stage of embryogenesis initiation), ‘globular embryos’ (stage of embryogenesis progression), and ‘cotyledonary embryos’ (differentiated embryos). As a commonly used test for evaluation of GSK-3β activity, we quantified ATP consumption levels following the kinase reaction using the Kinase-Glo™ assay, a luminescent assay for GSK-3 (Baki *et al.*, 2007). In the assay, the amount of ATP was proportional to the luminescent signal measured as RLUs and inversely correlated to GSK-3 enzymatic activity.

**Fig. 6.**
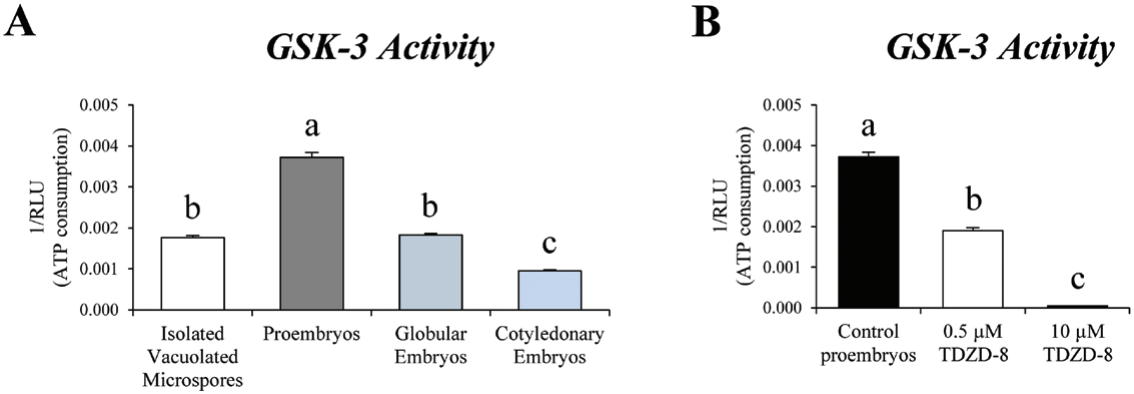
GSK-3 enzymatic activity during microspore embryogenesis in *B. napus* in control conditions and under TDZD-8 treatment. (A) The histogram represents GSK-3 enzymatic activity during consecutive developmental stages of microspore embryogenesis, quantified as ATP consumption (see the Materials and methods). (B) The histogram represents GSK-3 enzymatic activity at the proembryo stage in control cultures and cultures treated with 0.5 µM and 10 µM TDZD-8. Data represent the mean ±SEM. Different letters indicate statistically significant differences (*P*<0.05) obtained after ANOVA and subsequent Tukey HSD tests.

The assay of GSK-3 enzymatic activity showed an increase after embryogenesis induction, in proembryos, that reached >2-fold the kinase activity levels detected in vacuolated microspores before induction (Fig. 6A). As embryogenesis progressed, GSK-3 activity decreased, in globular embryos, and dropped again in cotyledonary embryos (Fig. 6A). To evaluate whether GSK-3 activity detected in microspore cultures was affected by TDZD-8 treatment, we assessed its enzymatic activity in samples treated with TDZD-8, at the proembryo stage and the concentration of 0.5 µM, which showed the highest effect over embryogenesis initiation efficiency (Fig. 3A). Treatment with TDZD-8 showed a significant decrease of GSK-3 enzymatic activity in proembryos compared with control cultures, with the inhibitor reducing ~50% of the activity detected in untreated cultures at the same stage (Fig. 6B). We also performed GSK-3 activity assays with culture samples treated with TDZD-8 at a higher concentration (10 µM), and the results showed that the activity was completely abolished, with 99.4% inhibition in microspore cultures at the proembryo stage (Fig. 6B).

### Expression patterns of brassinosteroid pathway genes during microspore embryogenesis

Given that BIN2, a master regulator of the BR pathway, is the homologue of mammal GSK-3β kinase, and that treatment with GSK-3β inhibitors enhanced microspore embryogenesis, we investigated the potential role of BRs in microspore embryogenesis. We analysed the gene expression patterns of key genes of the BR pathway; specifically, we analysed the BR biosynthesis gene *BnCPD*, BR signalling genes *BnBIN2*, *BnBES1*, and *BnBZR1*, and the BR catabolism gene *BnBAS1*, during microspore embryogenesis, at defined developmental stages.

**Fig. 7.**
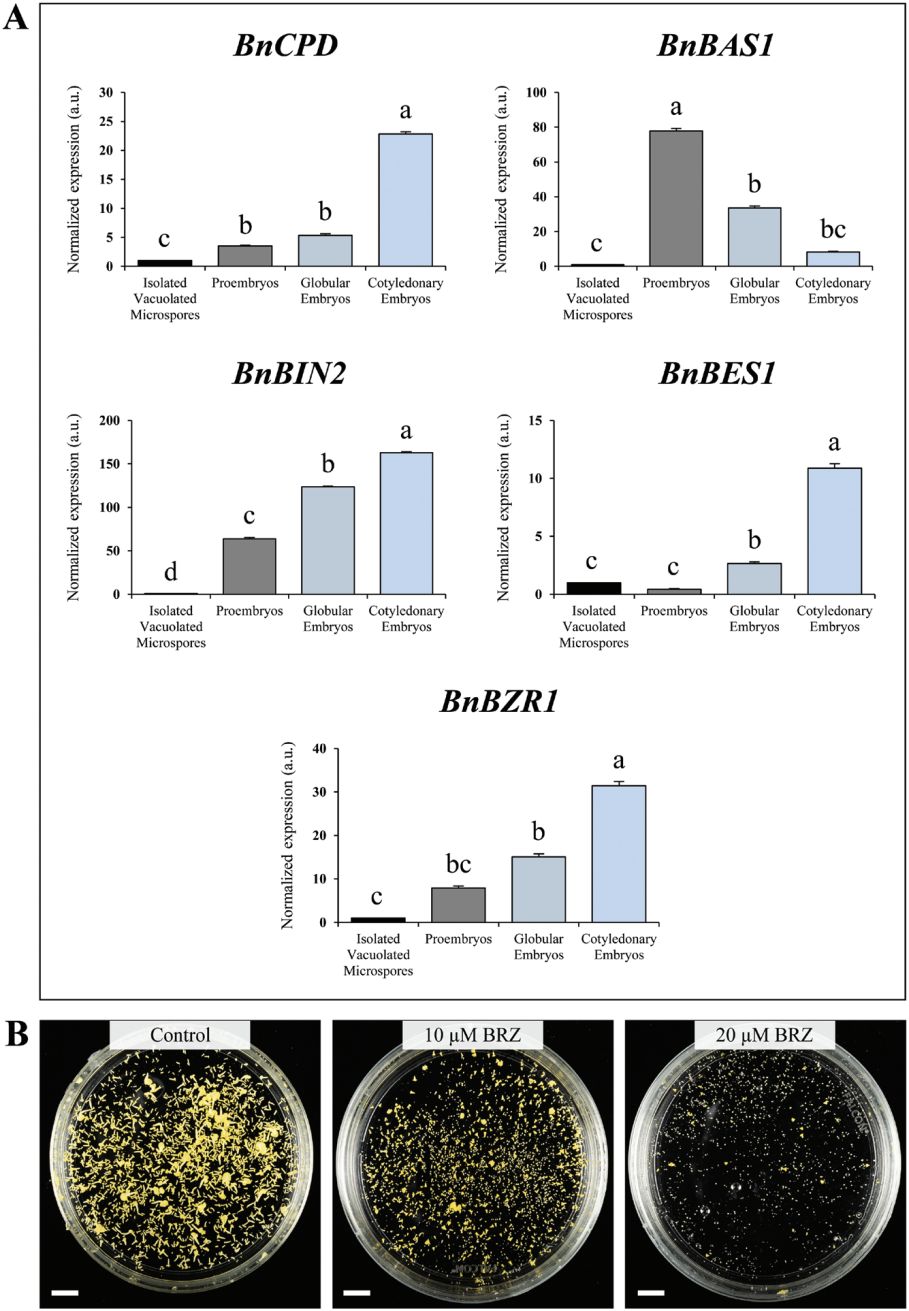
Expression patterns of genes of the brassinosteroid pathway and effect of brassinazole (BRZ) in microspore embryogenesis of *B. napus*. (A) RT-qPCR analysis of transcript accumulation of *BnCPD* (a BR biosynthesis gene), *BnBAS1* (a BR catabolism gene), *BnBIN2*, *BnBES1*, and *BnBZR1* (BR signalling pathway genes) normalized to vacuolated microspore levels. Data represent the mean ±SEM. Different letters indicate statistically significant differences (*P*<0.05) obtained after ANOVA and subsequent Tukey HSD tests. (B) Plates showing the microspore-derived embryos produced after 30 d in control, 10 µM BRZ-treated, and 20 µM BRZ-treated cultures. Scales bars, 10 mm.

RT-qPCR analysis of *BnCPD* revealed a significant increase of transcript levels after embryogenesis induction, at the proembryo stage, and a progressive increase throughout embryo development (Fig. 7A). Expression of *BnBIN2* followed a similar pattern of expression to *BnCPD*. *BnBIN2* transcripts significantly increased in the proembryo stage and during progression of microspore embryogenesis, in globular and mature cotyledonary embryos (Fig. 7A). BES1 and BZR1, two transcription factors of the BR signalling pathway, regulate expression of BR-responsive genes (Nolan *et al.*, 2020). Both *BnBES1* and *BnBZR1* expression levels significantly increased during embryo progression, reaching the highest levels in cotyledonary embryos (Fig. 7A). Interestingly, *BnBAS1* (a BR inactivation gene) showed an opposite profile; expression highly increased with microspore embryogenesis initiation, at the proembryo stage, while it decreased at later stages, in globular and cotyledonary embryos (Fig. 7A). Taken together, the expression profiles of the BR pathway genes indicated the progressive activation of BR biosynthesis and signalling after microspore embryogenesis induction, and during embryo differentiation.

### Effect of brassinazole, an inhibitor of BR biosynthesis, on microspore embryogenesis

To further confirm whether BRs have a role during stress-induced microspore embryogenesis, *B. napus* microspore cultures were treated with the known BR biosynthesis inhibitor BRZ. The results showed that embryo development was severely impaired in BRZ-treated cultures in comparison with control cultures (Fig. 7B), suggesting that BRs are required for embryo development during microspore embryogenesis in *B. napus*.

### Effect of the GSK-3β inhibitor TDZD-8 on overexpression of BR pathway genes at early and late stages of microspore embryogenesis

To evaluate whether TDZD-8 treatment had an effect on the BR pathway during microspore embryogenesis, we analysed expression levels of *BnCPD*, *BnBIN2*, *BnBES1*, *BnBZR1*, and *BnBAS1* in control and treated cultures. Since application of TDZD-8 on microspore cultures increased both proembryos (Fig. 3C) and embryos (Fig. 4C), cell extracts from both stages—early and late—were selected for RT-qPCR analysis in control and treated cultures.

**Fig. 8.**
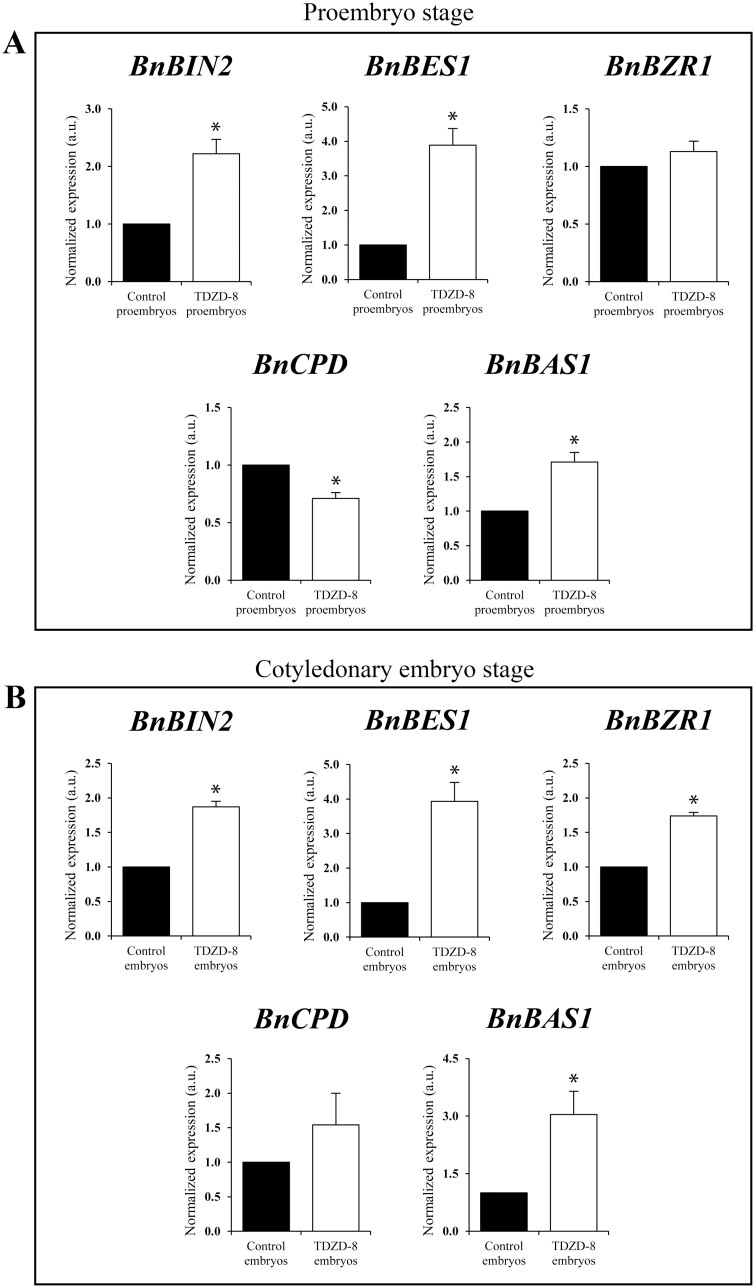
Effect of TDZD-8 on expression patterns of genes of the brassinosteroid pathway in microspore embryogenesis of *B. napus*. RT-qPCR analysis of transcript accumulation of *BnCPD*, *BnBAS1*, *BnBIN2*, *BnBES1*, and *BnBZR1* in microspore cultures of 4 d, at the proembryo stage (A), and after 30 d, at the cotyledonary embryo stage (B). Values are normalized to control culture levels. Data represent the mean ±SEM. Asterisks indicate statistically significant differences (*P*<0.05) obtained by Student’s *t*-test.

The expression analyses showed that BR signalling genes *BnBIN2*, *BnBZR1*, and *BnBES1* were up-regulated in proembryos treated with the GSK-3β inhibitor (Fig. 8A), while the BR biosynthetic gene *BnCPD* was down-regulated and the catabolic gene *BnBAS1* was up-regulated in proembryos treated with TDZD-8 (Fig. 8A), suggesting an effect of the compound on activating the BR signalling pathway, as well as limiting endogenous BR levels, to maintain BR homeostasis. The effect of TDZD-8 treatment on the same set of BR-related genes was analysed in cotyledonary embryos, after 30 d in culture. In treated embryos, changes in transcripts levels induced by TDZD-8, for most of the genes analysed (Fig. 8B), were similar to those in treated proembryos (Fig. 8A): TDZD8-treated embryos showed increased expression of BR signalling genes *BnBIN2*, *BnBZR1*, and *BnBES1*, as well as the BR catabolic gene *BnBAS1* (Fig. 8B). Expression of *BnCPD* did not show significant differences in treated embryos compared with control. The results suggested that GSK-3 inhibition by the small molecule TDZD-8 in microspore embryogenesis cultures enhanced the BR signalling pathway at early and late developmental stages.

### Effects of small molecule GSK-3β inhibitors during somatic embryogenesis in other crop and forest species

To further evaluate the possibility of extending these findings from *B. napus* to more distant species and different *in vitro* systems, three small molecule GSK-3β inhibitors (TDZD-8, VP3.15, and VP0.7) were applied to microspore and somatic embryogenesis cultures of *H. vulgare* and *Q. suber* as both species have well established *in vitro* embryogenesis systems, therefore they constitute model systems of the process in a cereal and a forest woody species, respectively.

**Fig. 9.**
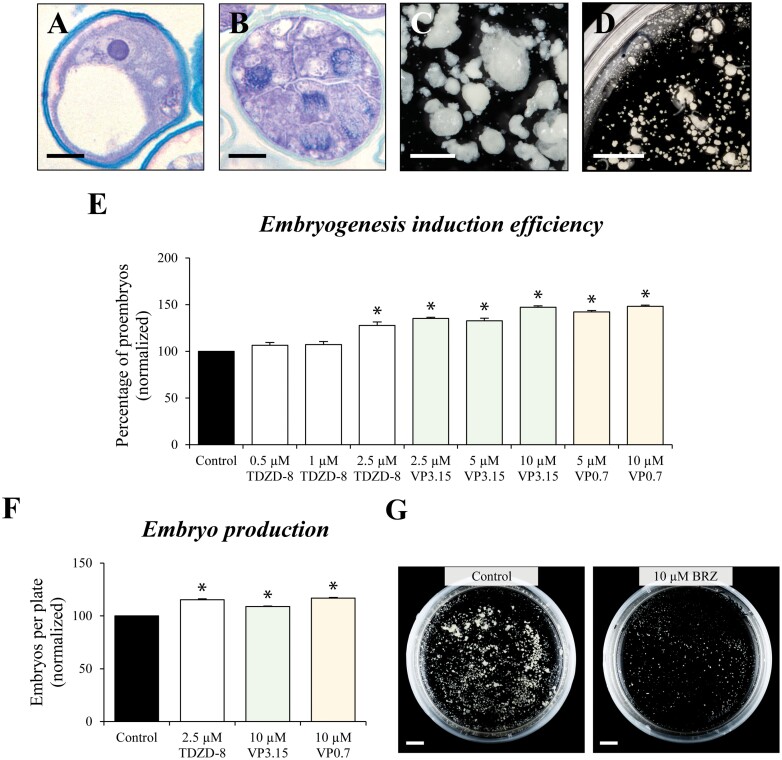
Effect of small molecule GSK-3β inhibitors and brassinazole on embryogenesis induction efficiency in microspore cultures of *H. vulgare.* (A–C) Main developmental stages of microspore embryogenesis in *H. vulgare*: (A) isolated vacuolated microspores, at culture initiation, (B) proembryos at 4 d of culture, and (C, D) globular, transitional and coleoptilar embryos. (C) Detail at higher magnification. (D) Panoramic view of a region of a 30 d culture plate. (E) Histogram with the quantification of proembryos, the first sign of embryogenesis initiation, in control cultures and cultures treated with the small molecules at different concentrations. (F) Quantification of total embryo production in control cultures and cultures treated with the inhibitors, after 30 d of culture. Columns represent the mean ±SEM. Values have been normalized to control culture (100%). Asterisks in (E) and (F) indicate statistically significant differences (*P*<0.05) between control and treated cultures obtained after Student’s *t*-test. (G) Plates showing the microspore-derived embryos produced after 30 d in control and 10 µM BRZ-treated cultures. Scales bars, 10 mm.

In *H. vulgare*, cold stress treatment of 4 °C was applied to induce microspore embryogenesis, as previously reported (Rodríguez-Serrano *et al.*, 2012). After induction, isolated microspores (Fig. 9A), cultured in liquid medium, were reprogrammed and proembryos were formed (Fig. 9B) as the first sign of embryogenesis initiation. During the subsequent days of culture, proembryos proliferated and followed a zygotic embryogenesis-like pathway of a monocot plant, through globular, transitional, and mature coleoptilar embryo stages (Fig. 9C, D). For each molecule, treatments were applied in microspore cultures of *H. vulgare*, firstly at the concentration that provided the best results in *B. napus* (Fig. 3C), specifically 0.5 µM TDZD-8, 2.5 µM VP3.15, and 5 µM VP0.7. Since we did not find significant differences compared with controls at this initial concentration with TDZD-8, treatments with higher concentrations were tested (1 µM and 2.5 µM TDZD-8, 5 µM and 10 µM VP3.15, and 10 µM VP0.7). Embryogenesis induction efficiency in control and inhibitor-treated cultures was assessed through proembryo quantification. The results of the quantification showed that all inhibitors used (TDZD-8, VP3.15, and VP0.7) significantly increased the percentage of proembryos in comparison with control cultures (Fig. 9E), the increase being from 27% to 47%, at slightly higher concentrations than in *B. napus*. Furthermore, we evaluated the effects of these GSK-3β inhibitors on embryo differentiation. For each small molecule, treatments at the concentration that provided the greatest increase of proembryos also showed a significant increase in embryo formation compared with control cultures (Fig. 9F).

Since inhibition of BR biosynthesis by BRZ impaired embryo differentiation during microspore embryogenesis in *B. napus*, we also wondered whether it could have similar effects on the monocot *H. vulgare*. After 30 d, numerous embryos were produced in control cultures, while BRZ-treated cultures showed a drastic reduction of embryo production (Fig. 9G), indicating that BR was also required for microspore embryogenesis in *H. vulgare*.

**Fig. 10.**
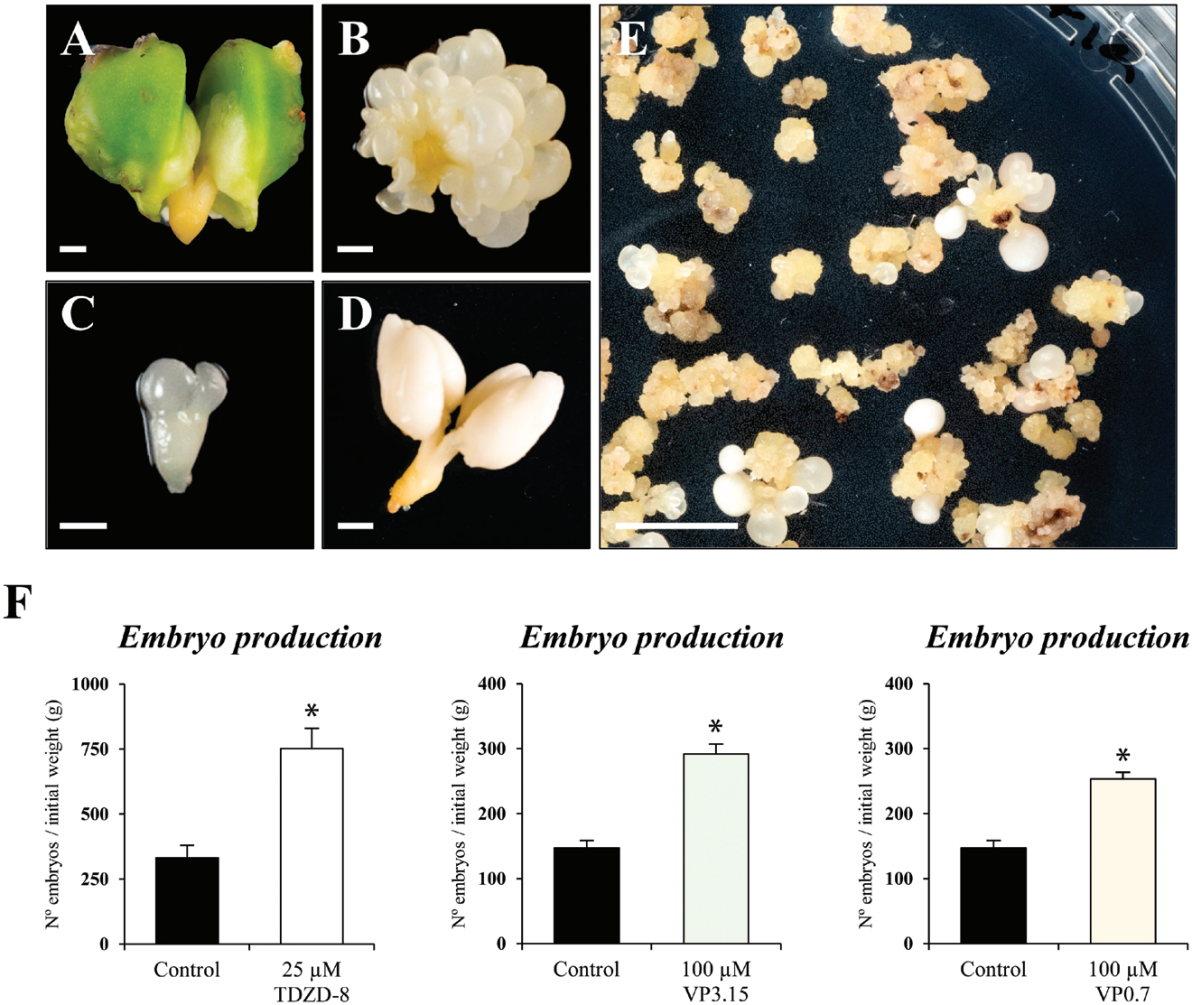
Effect of small molecule GSK-3β inhibitors on somatic embryogenesis of *Q. suber*. (A–E) Main stages of somatic embryogenesis in *Q. suber*: (A) immature zygotic embryo, initial explant before embryogenesis induction, (B) cluster of embryogenic masses, originated from the original explant after induction, (C) torpedo embryo, (D) mature cotyledonary embryo, and (E) panoramic view of a culture plate showing proliferating embryogenic masses and somatic embryos at different developmental stages. (F) Quantification of embryo production estimated as the number of cotyledonary embryos originated per gram of embryogenic masses in control cultures and cultures treated with the inhibitors. Data represent the mean ±SEM. Asterisks indicate statistically significant differences (*P*<0.05) between control and treated cultures by Student’s *t*-test. Scale bars, 1 mm in (A–C); 2 mm in (D); and 10 mm in (E).

The forest species *Q. suber* has well-established *in vitro* embryogenesis protocols constituting a model woody species for the process. In contrast to rapeseed and barley systems, which produced embryos directly from microspores, cork oak somatic embryogenesis produced embryos by an indirect pathway. Immature zygotic embryos, as the initial explant, subjected to induction conditions generate embryogenic masses, which proliferate and originate somatic embryos; furthermore, new embryos are also spontaneously formed from somatic embryo cells by secondary/recurrent embryogenesis (Testillano *et al.*, 2018). In the present study, *Q. suber* somatic embryogenesis was induced from immature zygotic embryos (Fig. 10A) as described (Testillano *et al.*, 2018). In the next weeks on induction medium, responsive cells switched their developmental programme and produced embryogenic masses (Fig. 10B) which further produced, asynchronically, new embryogenic masses and embryos, that could be found at different developmental stages such as globular, heart-shaped, torpedo (Fig. 10C), and mature cotyledonary embryos (Fig. 10D, E)

In this *in vitro* system, treatments with the GSK-3β inhibitors TDZD-8, VP3.15, and VP0.7 were performed. Since this system used solid culture medium, we applied concentrations 10× higher than in barley liquid media, as gelled media present lower diffusion and limited availability of their components than liquid media. Treatments were applied over isolated embryogenic masses, and embryo production was quantified as the number of cotyledonary embryos originated per gram of embryogenic masses at culture initiation, in control and treated cultures. As showed in Fig. 10F, the treatments with the three inhibitors led to a highly significant increase of somatic embryo formation in comparison with control cultures.

## Discussion

For many crop and forest species, low plant regeneration efficiency *in vitro* constitutes a key unsolved problem and a bottleneck in various breeding techniques such as gene editing, DH production, or micropropagation of elite genotypes. Despite the latest advances in understanding the mechanisms underlying cell reprogramming and embryogenesis, establishment of efficient *in vitro* protocols to regenerate plants from a cell or group of cells, to accelerate breeding programmes, is still a challenge for many cultivated plants. During the last years, in the biomedical field, substantial efforts have been made to develop more efficient and non-integrating methods for cell reprogramming. As a novel solution, cell-permeable small synthetic molecules, easy to apply and remove from cultures, have proved to be very useful for mammalian cell reprogramming and for generating the desired cell types (Ma *et al.*, 2017). Since increasing evidence has revealed that stem cells in plants and animals behave similarly (Olariu *et al.*, 2017), in the present study we have evaluated four small molecules, originally designed for therapeutic purposes, as potential chemical additives to promote plant cell reprogramming and regeneration in somatic embryogenesis cultures. The compounds are synthetic cell-permeable heterocyclic small molecules (mol. wt <500 Da) with inhibitory activity on mammalian GSK-3β, an inhibition that has been shown to stimulate cell reprogramming of human stem cells and *in vivo* neurogenesis enhancement (Morales-García *et al*., 2012). These chemicals have different chemical structure and mode of action, and had never been used in plants until the present report.

The results of the analyses showed similar positive effects on different *in vitro* embryogenesis systems and plant species with all the molecules tested. They increased embryogenesis efficiency in isolated microspore cultures of two crops, rapeseed and barley, as well as in somatic embryogenesis cultures of a forest tree, cork oak. The analyses performed revealed that the small molecules provided beneficial effects at both early and late stages of somatic embryogenesis. They were able to stimulate cell reprogramming and initial proliferation, increasing the percentage of proembryos formed (Figs 3C, 9E). Furthermore, the inhibitors also increased the final embryo production, at advanced stages (Figs 4C, 9F, 10F), with these embryos being of good quality, able to germinate (Fig. 4D) and convert into plantlets that further develop in a similar way to plants that are not treated with these molecules. Other types of molecules, with activity as epigenetic modulators, inhibitors of DNA methylation, histone H3K9 methylation, and histone deacetylases, have been shown to enhance cell reprogramming and proliferation in various plant *in vitro* systems (Li *et al.*, 2014; Solís *et al.*, 2015; Berenguer *et al.*, 2017), by the reduction of global epigenetic repressor marks. However, the presence of these epigenetic inhibitors during advanced developmental stages hindered further embryo differentiation (Solís *et al.*, 2015; Berenguer *et al.*, 2017). In the case of the GSK-3β inhibitors, they showed a positive effect over somatic embryogenesis at both early and advanced stages, which constitutes a great advantage in comparison with epigenetic modulators.

Several transcription factors, such as FUSCA 3 (FUS3), LEAFY COTYLEDON 2 (LEC2), and AGAMOUSLIKE 15 (AGL15), have been reported as key embryogenesis-specific markers and regulators of the initiation of somatic embryogenesis, being able to induce the process when ectopically expressed (Malik *et al.*, 2007; Horstman *et al.*, 2017; Méndez-Hernández *et al.*, 2019). Furthermore, endogenous auxin biosynthesis is activated with somatic embryogenesis induction (Ibañez *et al.*, 2020; Wojcik *et al.*, 2020), concomitantly with up-regulation of TRYPTOPHAN AMINOTRANSFERASE OF ARABIDOPSIS 1/TRYPTOPHAN AMINOTRANSFERASE-RELATED 2 (TAA1/TAR2), key enzymes of auxin biosynthesis (Yang *et al.*, 2012; Rodriguez-Sanz *et al.*, 2015; Pérez-Pérez *et al.*, 2019). In our study, expression patterns of *BnFUS3*, *BnLEC2*, and *BnAGL15* during microspore embryogenesis of *B. napus* showed a high up-regulation after embryogenesis induction in proembryos and globular embryos, dropping again at advanced stages (Fig. 5A). *BnTAA1* was also highly induced after embryogenesis initiation and during embryo development (Fig. 5A). Interestingly, treatments with the small molecule TDZD-8, as a representative compound of the group of molecules tested, significantly increased expression levels of these four markers of embryogenesis initiation at the stage of proembryos (Fig. 5B), indicating that the GSK-3β inhibitor had a positive effect on embryogenesis induction that resulted in higher expression of key molecular regulators of embryogenesis initiation and an increased number of proembryos.

The enzymatic assays performed have revealed for the first time the presence of GSK-3 activity in microspore cultures of *B. napus*, after microspore embryogenesis induction, in proembryos, with a progressive decrease of activity during embryo development (Fig. 6A). The assays performed with TDZD-8 revealed that the small molecule also inhibited GSK-3 activity in plants (Fig. 6B), as it did in humans (Martínez *et al.*, 2002).

Since the most relevant GSK-3 enzyme in plants is BIN2, a negative regulator of the BR signalling pathway (Nolan *et al.*, 2020), we wondered whether the BR pathway was active during the process in the absence of the small molecules. Genes of the BR biosynthesis pathway, *CPD*, and the BR signalling pathway, *BIN2*, *BES1*, and *BZR1*, showed similar expression profiles during microspore embryogenesis, with increasing levels of transcripts during consecutive stages of embryogenesis development (Fig. 7A). In contrast, the BR catabolic gene *BAS1* presented an opposite profile, with decreasing levels of expression throughout progression of embryogenesis. These expression profiles correlated with activation of the BR pathway (Nolan *et al.*, 2020), and clearly indicated that BR signalling was induced and progressively activated during microspore embryogenesis. Furthermore, the progressive decrease in GSK-3 activity during the process (Fig. 6A) also correlated with the progressive activation of the BR pathway, given that GSK-3–BIN2 is inhibited by the signalling cascade in response to the presence of BR (Nolan *et al.*, 2020).

The evidence supports the notion of a key role for hormonal regulation in plant somatic embryogenesis, with auxin playing a critical role in the reprogramming of somatic cells to embryogenesis (Fehér, 2015; Nic-Can and Loyola-Vargas, 2016). Previous reports have shown the key role of endogenous auxin in rapeseed and barley microspore embryogenesis induction and progression (Prem *et al.*, 2012; Rodríguez-Sanz *et al.*, 2015; Pérez-Pérez *et al.*, 2019). It has been demonstrated that induction of the auxin biosynthesis genes *TAA1/TAR2*, and increase of cellular auxin concentration, and its polar transport are required for cell reprogramming and embryo formation (Rodríguez-Sanz *et al.*, 2015; Pérez-Pérez *et al.*, 2019). However, much less is known about the involvement of other phytohormones such as BRs in the process. Several studies have reported that exogenous application of brassinolide (the most active BR) favoured somatic embryogenesis progression in some species (Pullman *et al.*, 2003; Ferrie *et al.*, 2005; Belmonte *et al.*, 2010; Chone *et al.*, 2018). However, before the present study, there were no reports on activation of the endogenous BR pathway during the process. Our results also showed that the inhibition of BR biosynthesis by BRZ in microspore cultures severely hindered embryo formation (Figs 7B, 9G), which additionally supports that endogenous BR has a key role during *in vitro* embryogenesis.

The treatment with the GSK-3β inhibitor TDZD-8 also affected the expression of BR-related genes. On the one hand, genes of the BR signalling pathway (*BIN2*, *BES1*, and *BZR1*) increased their expression in TDZD-8-treated cultures (Fig. 8), indicating that the inhibitor activated the pathway, mimicking the effect of BR. On the other hand, *CPD* (BR biosynthesis) did not change or decreased its expression with TDZD-8 treatment, while the catabolic gene *BAS1* was induced (Fig. 8), probably to maintain BR homeostasis in the presence of the inhibitor, which activated BR signalling, and mimicking the feedback effects of BR on these elements of the pathway (Nolan *et al.*, 2020). Interestingly, the same effect of TDZD-8 on expression of genes of the BR pathway was found at both proembryo and cotyledonary embryo stages of microspore embryogenesis, which suggested that BR has a role at early and late stages. Therefore, the inhibitor would enhance cell reprogramming and the embryogenesis initiation rate, increasing proembryo formation, and it would promote embryo development, increasing final embryo production. A previous report has shown the chemical inhibition of a subset of *Arabidopsis thaliana* GSK-3-like kinases by a synthetic molecule, bikinin, that acts as an ATP competitor (De Rybel *et al.*, 2009). Treatments with bikinin on Arabidopsis seedlings resulted in inhibition of BIN2 kinase and activation of the BR pathway, and led to constitutive BR responses such as an increase in hypocotyl length. This inhibitor also modified transcription levels of BR-related genes in the same way as exogenous brassinolide; specifically, it decreased expression of feedback-regulated BR biosynthetic genes, such as *CPD*, and increased transcription of BR signalling genes, such as *BIN2*, *BES1*, and *BZR1*, and BR-inducible genes, such as *BAS1* (De Rybel *et al.*, 2009). The small molecule TDZD-8 showed the same effects as bikinin on expression levels of BR-related genes in microspore embryogenesis, which strongly supports that GSK-3 inhibition by TDZD-8 results in activation of the BR signalling pathway.

Accumulated evidence indicates that endogenous hormones, mainly auxin and cytokinin, play an important role during *in vitro* plant development and somatic embryogenesis induction and progression (Periañez-Rodríguez *et al.*, 2014; Pérez-Pérez *et al.*, 2019; Testillano, 2019), data that open the door for targeting hormonal pathways as a potential biotech strategy to improve and accelerate crop plant regeneration and their application to breeding. However, until now, very low success has been achieved in developing practical protocols with chemical approaches for modulating hormone function to increase somatic embryogenesis yield in crop or forest plants. In our study, positive effects on somatic embryogenesis were found with four different small molecules that are potent inhibitors of GSK-3β in mammals, all of them with different chemical structures, showing IC_50_ values in the range of 1.6–4.4 µM (Martínez *et al.*, 2002; Palomo *et al.*, 2011, 2012; Pérez *et al*., 2011). Despite their different binding mode to inhibit mammalian GSK-3β, the results presented here provided evidence that all of the small molecules used promoted somatic embryogenesis induction and embryo formation in three different species, two crops (rapeseed and barley) and a forest tree (cork oak), as well as in three different somatic embryogenesis protocols, with liquid or solid medium, and by direct, indirect, or secondary/recurrent embryogenesis. The findings presented strongly suggest that a similar strategy using these inhibitors of mammalian GSK-3β could be extended to other species to increase plant cell reprogramming and embryo production yield. Further work will be required to identify the precise molecular target of the small molecules in somatic embryos of *B. napus* and the other species analysed; moreover, a deeper understanding of the mechanism of action of these molecules is required to exploit efficiently its promising potential applications in plant cell reprogramming and embryogenesis protocols. The rapid development in the design and synthesis of novel small compounds and chemical libraries for enzymatic targets will pave the way for new biotechnological strategies, by using small cell-permeable synthetic molecules, to enhance *in vitro* plant regeneration yield.

## Supplementary data

The following supplementary data are available at *JXB* online

Table S1. Data on expression stability of the reference gene *HEL.*

Table S2. Primer sequences used in this study.

erab365_suppl_Supplementary_Tables_S1-S2

## Data Availability

The data supporting the findings of this study are available from the corresponding author, P.S. Testillano, upon request.
