# Supplementary material for: Small molecule inhibitors of mammalian GSK-3β promote in vitro plant cell reprogramming and somatic embryogenesis in crop and forest species
Source: J Exp Bot. 2021 Aug 2;72(22):7808–25. doi: 10.1093/jxb/erab365 (PMC8664590; doi:10.1093/jxb/erab365)
Supplement: erab365_suppl_Supplementary_Tables_S1-S2 [file erab365_suppl_supplementary_tables_s1-s2.pdf]

Supplementary Table S1: Data on expression stability of reference gene *HEL*.

C_T value and standard deviation for *HEL* reference gene in the different experimental conditions

A) Different developmental stages of microspore embryogenesis

**Developmental stages**	**Mean C_T ± SD**
Vacuolated microspores	24.822 ± 0.193
Proembryos	26.625 ± 0.153
Globular embryos	25.145 ± 0.020
Cotyledonary embryos	26.262 ± 0.066

B) Proembryos in control and TDZD-8 treated cultures

**Proembryo conditions**	**Mean C_T ± SD**
Control culture	26.044 ± 0.095
TDZD-8 treatment	26.188 ± 0.082

C) Embryos in control and TDZD-8 treated cultures

**Embryo conditions**	**Mean C_T ± SD**
Control culture	27.862 ± 0.109
TDZD-8 treatment	27.595 ± 0.130

Supplementary Table S2. Primer sequences used for RT-qPCR assays.

Gene	Accesion number	Product size	Primer sequences (5' → 3')	
			Forward	*Reverse*
AtHEL	AT1G58050	61 bp	CCATTCTACTTTTTGGCGGCT	TCAATGGTAACTGATCCACTCTGATG
BnBIN2	Bra013341	171 bp	GCTGGTTGTGTTCTCGCTGA	ATGTGCCTTTATCTGCGGGA
BnBZR1	Bra015868	73 bp	GCTCTATGGTGCCGACTTCTC	GCGTTTGGAGACATCTGCTG
BnBES1	Bra031077	118 bp	GTCGTGGAGAGAGAGGGAGA	GTCACAATGCCTGGGAAGATT
BnCPD	Bra028751	72 bp	CCCAAACCACTTCAAAGACGC	GGACTTCTTGTTACCGAGTTGC
BnBAS1	Bra012046	126 bp	CATTTGGTCTTGGAGCCCGTA	TG GGT GCG TGT TGG TAA GTA
BnFUS3	DY012343.1	152 bp	GTCACCGCCACAGCCACTAC	CTCGACTGAGCCAAAACCCTCG
BnLEC2	DY021430.1	154 bp	TCAACTTCCATCACATCACCA	GCTCCTCGGAAATCTAAACGA
BnAGL15	DQ418546.1	150 bp	TGTGCTTGAAGATGGGTCGTGG	CAATGACGGCAACCTCAGCGT
BrTAA1	GBDX01015578.1	230 bp	CGTCACCCAATAACCCAGAC	CATCTTCTTTGCCACCTTCT
